# A Revision on the Effectiveness of Omega-3 Polyunsaturated Fatty Acids as Monotherapy in the Treatment of Major Depressive Disorder

**DOI:** 10.1155/2022/3801235

**Published:** 2022-11-16

**Authors:** Tommaso Accinni, Matteo Panfili, Lorenzo Tarsitani, Massimo Biondi, Massimo Pasquini, Annalisa Maraone

**Affiliations:** Department of Human Neurosciences, Sapienza University of Rome, Viale Dell'Universitá 30, 00185 Rome, Italy

## Abstract

**Background:**

Insufficient effectiveness and a difficult tolerability profile of antidepressant drugs for the treatment of major depressive disorder (MDD) have been reported, and polyunsaturated fatty acids (PUFAs) have been posited as reliable therapeutic alternatives. The present study investigated the efficacy of omega-3 PUFAs as monotherapy for MDD.

**Methods:**

Two well-trained reviewers independently looked at the most significant randomized clinical trials (RCTs) from the PubMed database regarding PUFAs' employment in MDD compared to placebo; “major depressive disorder” and “omega-3 fatty acids,” or “omega-6 fatty acids,” or “polyunsaturated fatty acids (PUFA),” or “*n* − 3 polyunsaturated fatty acids,” or “eicosapentaenoic acid (EPA),” or “docosahexaenoic acid (DHA)” were used as the medical subject keywords.

**Results:**

Of the initial 96 potential RCTs based on titles and abstracts, 82 studies did not meet the inclusion criteria and were excluded. Six studies were excluded from the remaining 14 after full text revision. Eight RCTs met all the inclusion/exclusion criteria without reporting clear evidence of PUFAs' effectiveness in the treatment of MDD.

**Conclusion:**

At present, there is no opportunity to recommend the use of omega-3 PUFAs monotherapy for the treatment of MDD, although their supplementation may be useful in some specific populations.

## 1. Introduction

Major depressive disorder (MDD) represents a severe mental disease with a prevalence of about 10% of the world's population, which influences general functioning and day-to-day life abilities. Several factors appear to contribute to MDD's heterogeneous etiological mechanisms [1–3]. Clinical research focused on defining effective treatments for both symptom improvement and prevention of recrudescence. Evidence has accumulated about efficacy of antidepressant drugs, in particular selective serotonin reuptake inhibitors (SSRIs), which nowadays is considered the first-line treatment of major depressive disorders (MDDs), as reported by all the most important clinical guidelines [4]. To date, clinicians must address remaining unresolved needs in relation to MDD treatments, considering that too often insufficient effectiveness and a difficult tolerability profile of antidepressant drugs may lead to poor adherence to treatments [5]. Indeed, it is well known that these drugs produce a clinically relevant response in only 60% of patients who undergo complete pharmacotherapy as established by protocols [6]. Moreover, MDD pharmacotherapy often has an impact on patients' everyday life, entailing several side effects such as sexual dysfunctions, gastrointestinal disorders, nausea, vomiting, and cardiac problems [7]. For these reasons, such drugs are not always well tolerated, and patients have dropped out of treatment protocols at a rate of about 30% [8]. Several alternative options for treating major depressive and mood disorders have been posited, among which polyunsaturated fatty acids (PUFAs) have attracted the attention of clinicians and researchers. PUFAs are a group of fatty acids considered essential because they cannot be synthesized by the human organism; thus, they are acquired through diet [9]. These molecules have a double carbon bond on the atom from the methyl end carbon (omega carbon) of the acyl chain and, for this reason, are defined as polyunsaturated; depending on the location of the double bond, they are categorized as omega-3 or omega-6 fatty acids [10]. The omega-3 fatty acids consist of docosahexaenoic acid (DHA) and eicosapentaenoic acid (EPA), which are derived from alpha-linolenic acid (ALA); seafood is considered the primary dietary source for these fatty acids, although they are also present in eggs, milk, plants, and vegetables [11]. On the other hand, plant oils such as sunflower, safflower, and corn oils are the main source of linoleic acid (LA), which is the omega-6 precursor and can be further metabolized to other omega-6 fatty acids such as gamma-linolenic acid (GLA) and arachidonic acid (AA) [12]. Interestingly, AA can then be further converted into prostaglandins and leukotrienes, which are responsible for proinflammatory effects. In contrast, omega-3 fatty acids reduce the synthesis of the proinflammatory mediators by acting as competitive inhibitors to omega-6 fatty acids [13]. PUFAs are fundamental components of cellular membranes that are involved in several aspects of neurotransmission; as neurotransmitters are associated with synthesis, release, and metabolism, they appear relevant for neurodevelopment and neural functioning as well [14–16].

Evidence has accumulated about the role of PUFAs omega-3 and omega-6 in preventing and treating mood disorders such as MDD; indeed, populations with higher dietary fish consumption showed lower rates of MDDs, as well as low rates of coronary disease mortality, cardiovascular disease mortality, stroke mortality, and general mortality [17, 18]. An inverse relationship between fish oil intake and the incidence of MDD has been previously described [19–27], and high seafood intake has been considered a protective factor against depressive episodes [28]. Consistent with these findings, patients with mood disorders presented lower levels of omega-3 PUFAs in blood and brain tissue samples [29, 30]. Similarly, low rates of DHA and total omega-3 PUFAs have been reported in postpartum depression [31], bipolar disorders were associated with low levels of DHA and AA [32] and anxiety disorders with low DHA and EPA as well [33]. Adult patients suffering from a major depressive disorder were reported to have lower omega-3 PUFA levels in their red blood cells and plasma [15]. Interestingly, a Cochrane study consisting of 25 randomized controlled trials, enrolling 1373 participants overall, assessed the efficacy of omega-3 fatty acids compared to placebo in major depressive disorder, with findings about small to moderate effects in reducing depressive symptoms [34, 35]. The study noted the efficacy of omega-3 PUFA supplementation in the treatment of depressive symptoms appeared to be conditioned by the proportion of DHA and EPA rates: combinations mainly composed of EPA (EPA > 50%, 60%, and 80% of the dose, respectively) showed significantly greater effectiveness compared to combinations mainly composed of DHA (DHA > 50%, 60%, and 80% of the dose, respectively), regardless of the PUFAs' employment as monotherapy or adjunctive strategies [36]. Moreover, evidence states that dosages ranging from 720 mg/d to 1000 mg/d and PUFA combinations with EPA rates at proportion of 60% and above appear to determine more significant effectiveness in treating depressive symptoms [36].

### 1.1. Omega-3 Fatty Acids Mechanism of Action on Depressive Symptoms

Although the exact biological mechanisms underlying the efficacy of omega-3 fatty acids (from now on, this term will be employed to refer to fatty acids clinically relevant in MDD treatment) in the treatment of depressive symptoms are still unclear, several hypotheses have been advanced. Omega-3 fatty acids take part in monoaminergic transmission regulation involving protein transcription; thus, low intake of omega-3 fatty acids entails lower levels of these fatty acids being present in the brain, along with an increased density of 5HT2 and D2 receptors in the frontal cortex [37–42]. A high intake of omega-3s is associated with serotoninergic metabolism, as already evidenced by the higher cerebrospinal fluid concentration of 5-hydroxy-indoleacetic acid (5-HIAA), which is a serotonin metabolite [17]. Moreover, the cerebrospinal concentration of 5-HIAA and somatotropin release appeared to increase with high omega-3 PUFA intake, in turn being associated with an improvement in depressive symptoms [43]. The anti-inflammatory capacity of omega-3 fatty acids has been suggested to explain their effect on MDD, which, in turn, is associated with an increase in proinflammatory markers [44]. The therapeutic effect of omega-3 fatty acids might be related to their anti-inflammatory and antioxidation activities, as already stated [45, 46]. Oxidative stress has been associated with mood disorders [47] through the evidence of increased reactive oxygen species (ROS) levels [48, 49]. Clinical improvements in patients with MDD seem to be favored by neuroplasticity and neurogenesis, as already stated, for therapeutic protocols involving antidepressants that appeared to increase neurogenesis in the hippocampus [50–52]. In addition, preclinical studies on animal models revealed that omega-3 fatty acids promote neurogenesis in the hippocampus [53–55] and play a role in neurotrophin modulation, likely mediating both neurogenesis and therapeutic effects [56–58].

## 2. Objectives

Although several studies have already addressed the efficacy of omega-3 and omega-6 fatty acids as adjunctive strategies to treat mood disorders and depressive symptoms, a lack of evidence emerges regarding the monotherapy employment of these fatty acids to treat major depressive disorders as an alternative to well-established therapeutic strategies according to international guidelines. Bearing this in mind, we sought to narratively review the literature to define whether polyunsaturated fatty acids such as omega-3 and omega-6 are effective in monotherapy for major depressive disorders, representing a potential and reliable alternative to the current therapeutic protocols.

## 3. Materials and Methods

We conducted a literature review of the most significant studies regarding the implementation of monotherapy with omega-3 fatty acids in treating major depressive disorder. We surveyed the PubMed database, searching for randomized clinical trials (RCTs) published starting from January 1990 to January 2022 on the abovementioned subject. We employed the following medical subject keywords: “major depressive disorder” and “omega-3 fatty acids,” or “omega-6 fatty acids,” or “polyunsaturated fatty acids (PUFA),” or “*n* − 3 polyunsaturated fatty acids,” or “eicosapentaenoic acid (EPA),” or “docosahexaenoic acid (DHA).” We subsequently looked at the most significant references from the abovementioned studies as well as relevant reviews and current controlled trials. We then included the most relevant randomized, double-blind, placebo-controlled studies (RCTs) that looked at the efficacy of omega-3 fatty acids as monotherapy for MDD in patients with an operationally diagnosed major depressive disorder according to DSM-IV or DSM-5 established criteria [59]. We excluded all studies that involved omega-3 fatty acids as add-on therapies, or that concerned samples characterized by nonspecific or nonclinically assessed depressive symptoms, or that were implemented on other mood disorders such as bipolar disorders, or where study participants had other neuropsychiatric comorbidities, or studies that did not reach a significant statistical relevance.

## 4. Results

**Figure 1:**
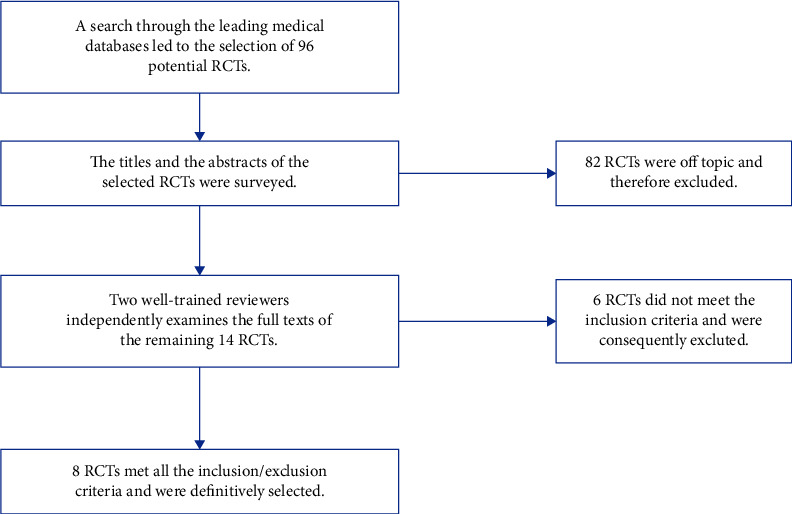
Flowchart showing the selection process for studies meeting the inclusion/exclusion criteria. RCTs, randomized clinical trials.

According to our inclusion criteria, we selected only studies of specific randomized clinical trials that investigated the efficacy of omega-3 fatty acids as monotherapy for MDD. Of the initial 96 potential RCTs, we first excluded 82 studies by reviewing the titles and abstracts. Of the remaining 14, we excluded 6 additional studies after two reviewers independently reviewed the full texts. Finally, 8 RCTs met all the inclusion/exclusion criteria (Table 1 and Figure 1).

The first study with this aim was conducted by Marangell et al. [60], who randomly assigned 35 patients with operationally diagnosed MDD to receive DHA 2 g/day or a placebo; patients were assessed by the Montgomery–Asberg depression rating scale (MADRS) [61] and the Hamilton depressive rating scale (HAM-D) [62]. They were enrolled with a score of MADRS >16 or HAM-D (28 items) >17, were not on psychotropic medication for at least two weeks, and were without neuropsychiatric or medical comorbidities. For a period of six weeks, 18 patients received DHA at a dose of 2 g/day, and 17 patients received placebo. Follow-up findings did not show significant differences between groups in regard to response rates, suggesting the absence of effectiveness of DHA monotherapy for adult outpatients with nonpsychotic major depression. This study represented the first placebo-controlled study employing omega-3 fatty acids as monotherapy for the treatment of unipolar major depressive episodes.

In 2008, Freeman and colleagues [63] investigated the efficacy of omega-3 fatty acid administration in a population of women with perinatal MDD. Fifty-nine women were randomized to 1.9 g/day EPA/DHA or placebo for eight weeks. Scores on the HAM-D and the Edinburgh postnatal depression scale (EPDS) [64] both showed a significant decrease for all groups, although some or no dose-related effect was observed.

A randomized control trial (RCT) was conducted by Su et al. in 2008 [65], comparing omega-3 fatty acids (3.4 g/d) with placebo in 36 pregnant women with a diagnosis of MDD assessed by the HAM-D and as secondary measures through the EPDS and the Beck Depression Inventory (BDI) [66]. After 8 weeks, subjects in the omega-3 group had significantly lower HAM-D scores and depressive symptoms, showing that omega-3 fatty acids may have therapeutic benefits in treating depression during pregnancy.

However, the opposite results appeared in a study conducted in the same year in women with major depression during the perinatal period. Twenty-six subjects were recruited and received either fish oil or a placebo for six weeks. The results suggested that there was no benefit for omega-3 fatty acids over placebo in treating MDD during the perinatal period [67].

Jazayeri et al. [68] sought to compare the therapeutic effects of EPA, fluoxetine, and their combination in MDD. Sixty outpatients who had received the diagnosis of MDD based on DSM-IV criteria and who had scored >15 in the 17-item HAM-D were randomly assigned to receive either EPA (1 g) or fluoxetine (20 mg) or their combination daily for 8 weeks. The combination of EPA and fluoxetine was significantly more effective than the administration of only EPA or fluoxetine from the fourth week of treatment; fluoxetine and EPA appeared to be equally effective in improving depressive symptoms, with response rates (>50% decrease in baseline HAM-D) of 50%, 56%, and 81%, respectively, in the fluoxetine, EPA, and combination groups. A synergistic interaction between EPA and fluoxetine was somehow suggested.

In 2015, Mischoulon et al. [69] compared the efficacy of EPA and DHA as monotherapies for MDD by implementing a 2-site, placebo-controlled, randomized, double-blind clinical trial. One hundred and ninety-six participants with a diagnosis of MDD based on DSM-IV criteria and with a baseline score at the HAM-*D*-17 of ≥ 15 were initially enrolled, but only 154 participants completed the program. Participants were randomly assigned to receive an oral treatment with oral EPA-enriched*n* − 3 1000 mg/d, DHA-enriched*n* − 3 1000 mg/d, or a placebo for 8 weeks. All the recruited groups showed significant improvement in depressive symptoms as evaluated by the outcome measures (the HAM-D-17, the 16-Item Quick Inventory of Depressive Symptomatology-Self Report (QIDS-SR-16) [70], and the Clinical Global Impression-Severity scale (CGI-S) [71]), but no significant differences were found between groups receiving omega-3 fatty acids and the placebo group. Response and remission rates for recruited groups were 40%–50% and 30%, respectively, but no significant differences between them were observed. Neither EPA-enriched nor DHA-enriched combinations appeared significantly more effective than a placebo for the MDD treatment.

Chang et al. [72] sought to investigate the efficacy of omega-3 fatty acids for the treatment of MDD in a population of individuals with cardiovascular comorbidity. Fifty-nine patients were randomly assigned to receive either omega-3 fatty acids (2 g per day of eicosapentaenoic acid, EPA, and 1 g of DHA) or a placebo for 12 weeks. They underwent an assessment based on the HAM-D, the BDI, the electrocardiogram, and the blood biochemistry, both at baseline and endpoint. The authors did not observe any significant difference between groups concerning depressive symptom improvement as described by the psychometric scores. However, once stratified for depression severity, the omega-3 fatty acid supplementation appeared to improve core depression symptoms [73] in the very severe MDD group [72].

Gabbay et al. sought to investigate the efficacy of the employment of omega-3 fatty acids as monotherapy for MDD in a sample of adolescents. They found that omega-3 fatty acids did not lead to significant improvement compared to placebo regarding any clinical feature of MDD, including symptoms' severity, levels of anhedonia, irritability, and suicidality. No significant differences resulted between groups in terms of response rates either [74].

## 5. Discussion

Starting from the literature that was accumulated about the minimal evidence of omega-3 fatty acid efficacy as reliable treatments for mood disorders and depressive symptoms, we decided to strictly include in our review design only findings based on studies that considered omega-3 fatty acids as monotherapy for major depressive disorder. We aimed to shed light on these relatively new treatment strategies, questioning whether to consider them reliable alternatives to common antidepressant drugs in the framework of MDD treatment approaches. To our knowledge, the present narrative review represents the first attempt to recognize and collect the most relevant evidence reported in the literature about the abovementioned clinical question. What emerges from our review is the lack of clear evidence of the efficacy of omega-3 fatty acids employment as monotherapy for MDD, considering the contradictory nature of reported findings. Indeed, studies do not seem to reach a common agreement over the opportunity of fatty acids' clinical employment for such a severe mental illness as major depressive disorder, and most of the effects of their administration were reduced once publication bias was corrected. However, it is important to note that several methodological issues may have affected previous studies and should be addressed to clarify such clinical hypotheses: studies have varied concerning specific omega-3 fatty acids employed, sample sizes, duration of therapies, posology of administered combinations, and the employment of omega-3 fatty acids as monotherapy or adjunctive therapies [75]. Although all the recruited studies met the established inclusion criteria, different variables have been considered, such as the studied populations, the psychometric rating scales that have been employed, and the administered formulations and dosages of omega-3 fatty acids. For instance, we observed that the use of omega-3 fatty acids in monotherapy for MDD appeared to be significantly effective in special populations such as women with perinatal MDD [63], pregnant women [65], and individuals with cardiovascular comorbidity and severe MDD [72]. Other differences in study designs are nested in the drug combinations or formulations that have been employed; for example, Jazayeri et al. [68] have focused on the synergistic interaction between omega-3 fatty acids and fluoxetine.

Thus far, no common agreement about dosage and duration of omega-3 fatty acid administration has been reached to demonstrate their potential efficacy in MDD treatment, although 1 g/day of EPA or combinations mainly composed of EPA demonstrated higher efficacy than combinations mainly composed of DHA, consistent with previous literature [36]. The fact that specific combinations of fatty acids such as EPA and DHA might differ in their effectiveness in treating MDD should warrant greater attention from clinical researchers.

Our findings seem partially consistent with those suggesting that omega-3 combinations mainly composed of EPA appear more effective, both in monotherapies and adjunctive treatment strategies. In this regard, concerning the higher effectiveness of the 2 : 1 ratio EPA/DHA formulation, only Su et al. [65] reported a significant improvement in depressive symptoms in a population of women with MDD. The other two studies employing a 2 : 1 ratio EPA/DHA formulation [72, 74] did not confirm such results. It is worth noting that the last two studies were conducted in special populations, such as adolescents and patients with cardiovascular comorbidities (Table 1). Moreover, these studies used different psychometric rating scales to investigate specific clinical dimensions. Su et al. used the HDRS, EPDS, and BDI scales, which are particularly focused on categorical depressive symptoms, whilst Gabbay et al. employed the CDRS-R scale, a tool tailored to children and adolescent populations, and the CGI scale, focusing on symptoms' severity outcomes. Therefore, it cannot be ruled out that these different study designs may have affected the results and that heterogeneity in omega-3 combinations may have led to contradictory findings with regard to their effectiveness. In the present review, we sought to accurately describe the different omega-3 combinations employed in the recruited RCTs (Table 1). Therefore, considering our findings, we did not report clear recommendations about the employment of specific omega-3 combinations as monotherapy for MDD, but, as previously mentioned, other studies are required to shed light on this topic. Bearing this in mind, it should be noted that the trial conducted by Marangell et al. [60] involved a treatment intervention with only DHA and not EPA or other omega-3 fatty acids, likely leading to conditioning findings. Other methodological concerns may have influenced previous clinical findings, such as the lack of coherence in the diagnosis and the criteria definition to enroll participants who would have been assessed. In order to address this aspect, we sought to limit our review to RCTs and studies that recruited people operationally diagnosed with major depressive disorders, according to DSM-IV or DSM-5 criteria. Therefore, we should notice that the study conducted by Tajalizadekhoob et al. [76] was implemented on a sample of elderly individuals whose depressive symptoms were assessed through the scoring of the Geriatric Depression Scale-15 (GDS-15) [77] and not based on operational criteria. Moreover, from this study, it appears that participants' depression was significantly improved by omega-3 fatty acids intake when patients were not under antidepressant therapy, likely indicating a less severe degree of depression. Indeed, no significant differences in GDS-15 appeared between groups when considering participants taking antidepressant drugs: the effect of omega-3 on depressive symptoms of elderly people may be partially explained by the average low intake of fatty acids due to the poor diet this population is exposed to.

A larger effect of fatty acids in samples of individuals taking antidepressant drugs has been observed, likely suggesting a synergistic effect between them and omega-3 fatty acids [68, 78]. It has been posited that fatty acids may biologically modulate both the interaction between neuronal membranes and antidepressants and the inflammatory pathways activated in depressive states [78–81]. Other potential mechanisms concern the evidence for omega-3 fatty acids' role on serotonergic neurotransmission [82] and the p-glycoprotein mediated enhancement of antidepressant transport across the blood-brain barrier [83].

Overall, findings from selected studies showed a lack of clear evidence for the efficacy of the employment of omega-3 fatty acids as monotherapy for MDD, despite some empirical use and biological evidence, a hypothesis that is corroborated by relevant meta-analyses and systematic reviews involving both studies that used omega-3 fatty acids in monotherapy and in augmentation treatment. In 2012, Bloch and Hannestad [35] conducted a meta-analysis involving 13 RCTs on 731 participants with the aim of comparing omega-3 fatty acids to placebo in patients with moderate or mild depressive symptoms at baseline. In 7 RCTs, omega-3s were used as monotherapy, and in 6 RCTs, they were used as an augmentation treatment. They found a small, nonsignificant benefit of omega-3 fatty acids for the treatment of MDD. This meta-analysis included studies involving self-rating scales instead of diagnostic and structured interviews, likely affecting results. Interestingly, another meta-analysis conducted by Yang et al. in 2015 [84] reviewed 8 RCTs involving 367 participants receiving a combination of DHA and EPA compared to a placebo for the short-course treatment of depression in women: 6 RCTs investigated the efficacy of the combination administered as monotherapy and 2 RCTs as augmentation therapy. The authors found that the combination of EPA and DHA appeared to show a beneficial effect on depressed mood in women with MDD compared with placebo. It should be noted that the heterogeneity of the trial designs that have been incorporated into the main meta-analyses may partially explain their contradictory findings. These studies often involved RCTs investigating the effectiveness of omega-3 fatty acids in augmentation strategies and not in monotherapy or including subthreshold depressive symptoms without meeting criteria for a MDD diagnosis. However, in a recent review [85], the expert consensus of the International Society for Nutritional Psychiatry Research (ISNPR) suggested the use of omega-3 PUFAs for the treatment of MDD in specific patient subgroups like pregnant women, children, and the elderly. Interestingly, it has been suggested that in adolescents and young adults, the use of omega-3 fatty acids for MDD treatment may represent a reliable strategy likely to avoid major side effects of traditional drugs [85]. Although patients affected by MDD at different ages may show specific symptoms, it has been reported that the efficacy of omega-3 fatty acids does not appear to vary with regard to the age of the recruited samples [86]. The employment of omega-3 fatty acids for prevention in populations at high risk for depression has been suggested as well. Moreover, omega-3 fatty acid efficacy has been reported in populations of patients with depressive disorders and medical comorbidities such as cardiovascular, inflammatory, and metabolic diseases; it may be suggested that the well-establishedanti-inflammatory activities of omega-3 fatty acids would play a relevant role in this respect, likely reducing inflammatory factors, which in their turn appear associated with depression [15, 72, 87]. Some authors suggested a role for diets with low omega-3 fatty acid intake in increasing the risk of the development of depressive symptoms in high-risk populations [69]. Indeed, the findings that support the strength of omega-3 fatty acids efficacy in MDD treatment have appeared to decrease according to more recent studies in the literature where the latter have been implemented in samples and populations adopting increasingly balanced diets with already higher omega-3 fatty acids intake compared to the past. This factor likely influenced findings, and both the relevant confounders' variables and the socioeconomical background should be addressed in order to arrive at a valid assessment of the omega-3 fatty acids efficacy. For example, as already reported, the association of low fish intake with an increased incidence of mood disorders was the primary reason to investigate the efficacy of omega-3 fatty acids in the treatment of these mental illnesses, thus suggesting a confounding role for the basic economic status with which diet likely appears associated [88]. Similarly, higher efficacy of omega-3 fatty acids was associated with studies, with high heterogeneity likely influencing the effect sizes to the detriment of the real efficacy of omega-3 fatty acids [35]. To note, the tolerability of omega-3 fatty acids has been previously established, and their administration has been associated with few impairing side effects such as fishy belching, flatulence, and diarrhea. However, unclear evidence about long-term administration effects has been provided given the evidence of omega-3 fatty acids' predisposition to oxidative degradation, which may potentially represent a late adverse effect [89]. More studies are required in this field.

## 6. Conclusions

We reviewed the most relevant RCTs investigating the employment of omega-3 fatty acids as monotherapy in MDD treatment, and we did not find any clear evidence of efficacy for such therapeutic strategies. Bearing our findings in mind, we suggest that the use of omega-3 PUFAs as a monotherapy for the treatment of MDD is not yet supported by significant evidence of effectiveness, and more studies are likely required to shed light on this topic. However, omega-3 fatty acids supplementation or a diet rich in omega-3, thanks to their quite safe profile, may be suggested in some specific populations to prevent or reduce depressive symptoms. Overall, the therapeutic effect of omega-3 fatty acids in major depressive disorder is lacking, and, to date, opportunity costs and ineffectiveness do not allow for their consideration as a valid alternative in treatment programs.

## Figures and Tables

**Table 1:** Principal RCTs on the effectiveness of omega-3 fatty acids as monotherapy in the treatment of MDD.

Study	Year	Study duration	Omega-3formulation vs. placebo	Formulation	Subjects, *n*(*I*/*C*)	Population	Diagnosis	Rating	Results
Marangell et al.	2003	6 weeks	2 g DHA	DHA	35 (18/17)	Adult outpatients	MDD	MADRS, HDRS	−
Freeman et al.	2008	8 weeks	1.1 g EPA + 0.8 g DHA	Omega-3	59 (31/28)	Women	MDD	HDRS, CGI, EPDS	−
Su et al.	2008	8 weeks	2.2 g EPA + 1.2 g DHA	Omega-3	36 (18/18)	Women	MDD	HDRS, EPDS, BDI	+
Rees et al.	2008	6 weeks	6 g fish oil soft gelatin (27.3% DHA, 6.9% EPA)	Omega-3	26 (13/13)	Women	MDD	HDRS, MADRS, EPDS	−
Jazayeri et al.	2008	8 weeks	1 g EPA/fluoxetine	EPA/fluoxetine	60 (20/20/20)	Adult outpatients	MDD	HDRS	+
Mischoulon et al.	2015	8 weeks	EPA/DHA (EPA enriched: 1.06 g EPA + 274 mg DHA; DHA enriched: 900 mg DHA + 180 mg EPA)	EPA/DHA	196 (66/65/65)	Adult outpatients	MDD	HDRS, QIDS-SR-16, CGI-S	−
Gabbay et al.	2018	10 weeks	2.4 g EPA + 1.2 g DHA	Omega-3	51 (24/27)	Adolescents	MDD	CDRS-R, CGI	−
Chang et al.	2020	12 weeks	2 g EPA + 1 g DHA	Omega-3	59 (30/29)	Patients with cardiovascular comorbidity	MDD	HDRS, BDI	−

BDI, Beck Depression Inventory; CDRS-R, Children's Depression Rating Scale-Revised; CGI, clinical global impression; DHA, docosahexaenoic acid; EPA, eicosapentaenoic acid; EPDS, Edinburgh postnatal depression scale; HDRS, Hamilton depression rating scale; I/C, intervention/control; MADRS, Montgomery–Asberg depression rating scale; MDD, major depressive disorder; PUFAs, polyunsaturated fatty acids; QIDS-SR-16, 16-Item Quick Inventory of Depressive Symptomatology-Self Report; RCTs, randomized clinical trials; +, significant results; −, nonsignificant results.

## Data Availability

The data that used to support the findings of this study are available from the corresponding author upon request.
